# Diagnosis of rejection following heart transplantation: diving into the future

**DOI:** 10.3389/frtra.2025.1693821

**Published:** 2026-01-22

**Authors:** Shaline Rao, Syed Zain Ali, Arushi Singh, Mittal Rana, Mohamed Moussa, Kinza Ahmed, Stephanie Golob, Lauren Cusumano, Alana Harrington, Andrew Wang, Sanjay Chandrasekhar, Amit Alam

**Affiliations:** 1 Division of Cardiology, Department of Medicine, New York University Long Island Grossman School of Medicine, NYU Langone Health, Mineola, NY, United States; 2 New York University Long Island Grossman School of Medicine, NYU Langone Health, Mineola, NY, United States

**Keywords:** heart transplant (HTx), rejection, diagnosis, non-invasive surveillance, heart

## Abstract

Since the standardization of the grading system for pathologic diagnosis of antibody-mediated and acute cellular rejection, endomyocardial biopsy has remained the gold-standard. However, biopsies are invasive, costly, and limited by sampling error. As such, adjuvant non-invasive methods including cardiac biomarkers, imaging including cardiac magnetic resonance and echocardiography, and donor-specific antibodies and non-HLA antibodies have been traditionally used. However, all these techniques are limited by either sensitivity or specificity. More recently, there has been a shift to other contemporary non-biopsy surrogate markers for rejection surveillance including donor-derived cell free DNA, gene expression profiling, and messenger RNA and micro-RNA in tissue. Herein we review the methods currently utilized to diagnose rejection and their limitations. We find that while there have been significant advancements in technology and non-invasive techniques, no current method alone adequately diagnoses rejection (Central Image). Thus, future studies are warranted to investigate new strategies involving a multi-modal approach that incorporates non-invasive diagnostic methods and personalized medicine to monitor postoperative progression in heart transplant patients.

## Evolution of definition of rejection

In the 1990s the International Society of Heart and Lung Transplantation (ISHLT) standardized the grading system for the pathologic diagnosis of rejection in cardiac biopsies (1). This was necessary to facilitate communication between clinicians and centers. Acute cellular rejection (ACR) was graded based on evidence and extent of myocyte damage. Surveillance for humoral rejection was not yet recommended, it was defined as evidence of positive immunofluorescence, vasculitis, or severe edema in the absence of cellular infiltrate. In 2004 the ISHLT statement revised the grading system to reflect field advances in immunosuppression and a shift in the clinical response to milder forms of rejection. The 2004 update recognizes acute humoral rejection as a clinical entity, however at this time it's significance remained controversial (2) (Table 1). In 2010 a consensus conference sponsored by the ISHLT convened to advance the understanding of antibody mediated rejection (AMR). Contrary to the 2004 statement, this led to the recommendation for routine screening of AMR and included the use of specific staining, and assessment of specific circulating antibodies (3). In 2013 the ISHLT working formulation further expanded on the diagnostic criteria and grading of pathological AMR, proposing evaluation of markers of complement activation, endothelial injury, thrombotic environment, and intravascular macrophages (3). In the 2023 ISHLT guidelines for the care of heart transplant recipients' recommendations are made for routine post-transplant DSA monitoring (4) (Table 2).

**Table 1:** Evolution onf ISHLT consensus acute cellular rejection definitions.

Year	ACR grade	Definition
1990	0	No rejection
	1, Mild	A - Focal: Focal perivascular and/or interstitial infiltrate without myocyte damage
		B - Diffuse: Diffuse infiltrate without myocyte damage
	2, moderate (focal)	One focus of infiltrate with associated myocyte damage
	3, moderate	A - Focal: Multifocal infiltrate with myocyte damage
		B - Diffuse: Diffuse infiltrate with myocyte damage
	4, severe	Diffuse, polymorphous infiltrate with extensive myocyte damage +/- edema, +/- hemorrhage, + vasculitis
2004	0 R	No rejection
	1 R, mild	Interstitial and/or perivascular infiltrate with up to 1 focus of myocyte damage
	2R, moderate	Two or more foci of infiltrate with associated myocyte damage
	3R, severe	Diffuse infiltrate with multifocal myocyte damage +/- edema, +/- hemorrhage, +/- vasculitis

**Table 2:** Evolution of ISHLT consensus antibody- mediated rejection definitions.

Year	AMR grade	Definition
1990	None	Humoral rejection (positive immunofluorescence, vasculitis or severe edema in absence of cellular infiltrate) recorded as additional required information
2004	AMR 0	Negative for acute antibody-mediated rejection: - No histologic or immunopathologic features of AMR
	AMR1	Positive for AMR: - Histologic features of AMR - Positive immunofluorescence or immunoperoxidase staining for AMR (positive CD68, C4d)
2010	pAMR 0	Negative for pathological AMR: - Both histological and immunopathologic studies are negative
	pAMR 1(H+)	Histopathologic AMR alone: - Histological findings present and immunopathologic findings negative
	pAMR (I+)	ImmunopathologicAMR alone: - Histological findings negative and immunopathologic findings positive
	pAMR 2	Pathological AMR: - Both histological and immunopathologic findings are present
	pAMR 3	Severe pathological AMR: - Severe AMR with histopathologic findings of interstitial hemorrhage, capillary fragmentation, mixed inflammatory infiltrates, endothelial cell pyknosis and/or karyorrhexis, and marked edema

**Central Image:**
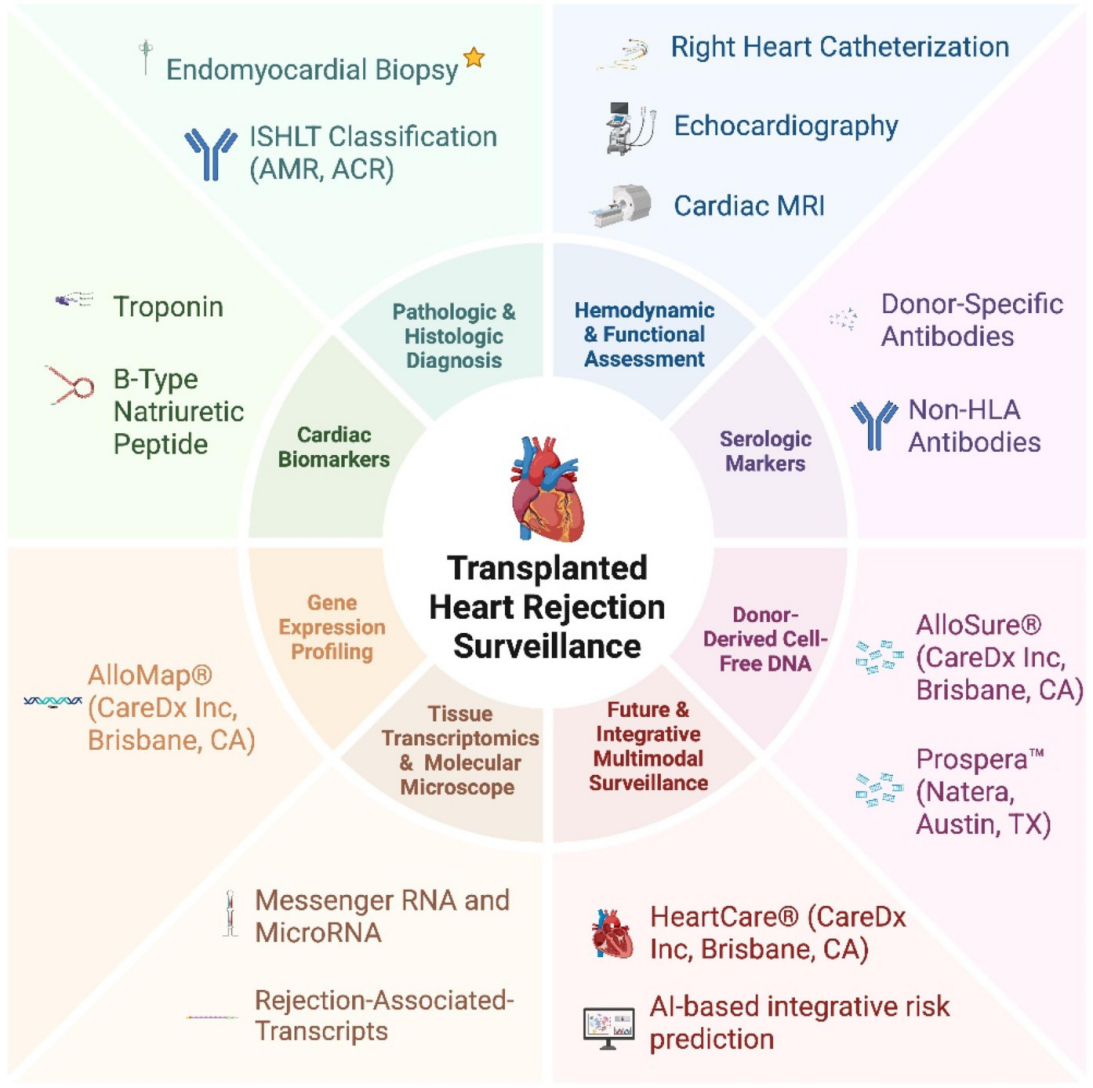
Heart transplant rejection surveillance modalities.

## The traditional endomyocardial biopsy

Endomyocardial biopsy (EMB) has long been the gold standard for detecting allograft rejection following heart transplantation. The transvalvular approach, using a bioptome, was first introduced in the 1960s by Drs. S Sakakibara and S. Konno in Japan, initially intended for diagnosing myocarditis and cardiomyopathies (5). Roughly a decade later, the Stanford group developed a flexible bioptome capable of serial sampling, allowing EMB to be integrated into routine post-transplant surveillance protocols (6). By enabling direct histopathological assessment of myocardial tissue, EMB provided a critical window into the immune response within the cardiac allograft, revolutionizing post-transplant care. Following the advent of heart transplantation, particularly after the development of cyclosporine-based immunosuppression in the 1980s, EMB gained prominence. The method allowed clinicians to detect acute cellular rejection and tailor immunosuppressive therapy, markedly improving graft survival and patient outcomes (6).

Despite its clinical utility, traditional EMB has notable limitations (Table 3). One significant challenge is its invasive nature, which carries inherent risks such as bleeding, arrhythmias, and infection. Although generally low per biopsy, these risks accumulate with repeated sampling, a common necessity especially in the first-year post-transplant when most rejection episodes occur. Repetitive biopsies have been associated with tricuspid valve regurgitation due to multiple crossings of the valve with the bioptome (6). This has been reported in up to 23% of patients in some studies, potentially leading to right heart dysfunction over time (7).

**Table 3:** Limitations of endomyocardial biopsy.

Limitations	Description
Invasiveness	Requires catheterization and bioptome use, increasing risks of bleeding, arrhythmias, and infection
Tricuspid Valve Trauma	Repeated biopsies can cause trauma to the tricuspid valve, leading to regurgitation and potential RV dysfunction
Sampling Error	Biopsy samples only localized tissue areas, risking false negatives if rejection occurs in unsampled regions
Interobserver Variability	Differences in histopathological interpretation may lead to inconsistent grading and clinical decisions
Resource Intensity	Requires catheterization labs, specialized personnel, and multiple procedures, increasing logistical burden
Cumulative Patient Risk	Risks of complications accumulate with repeated biopsies, especially early post-transplant, increasing patient morbidity

Another notable limitation is the potential for sampling error. Because EMB samples a small portion of the right ventricular septum, rejection in unsampled areas may go undetected, resulting in false negative biopsies. Additionally, interobserver variability in histopathologic interpretation can lead to inconsistent grading of rejection severity, impacting clinical decisions (8, 9). In addition, the costs and logistical demands of serial EMB, including the need for catheterization lab resources and pathology support further highlight the need for complementary diagnostic modalities (10).

While endomyocardial biopsy has limitations in terms of invasiveness, cost, and diagnostic accuracy, its role in heart transplant rejection diagnosis and guiding immunosuppressive therapies is undeniable (11). It has been instrumental in shaping our understanding of cardiac allograft rejection and remains foundational in the development of both immunosuppressive strategies and emerging diagnostic technologies. As the field continues to advance, EMB serves as the benchmark against which newer, less invasive approaches are measured (12).

## Mimickers of rejection

Graft dysfunction encompasses a range of immunologic and non-immunologic complications that progressively impair allograft performance. Apart from ACR and AMR, non-rejection causes of graft dysfunction include primary graft dysfunction (PGD), cardiac allograft vasculopathy (CAV) and infections (Table 4).

**Table 4:** Reasons for graft dysfunction without rejection.

Category	Cause	Mechanism	Clinical impact
Primary Graft Dysfunction (PGD)	Ischemia-reperfusion injury, donor/recipient risk factors	Myocardial stunning, endothelial dysfunction, inflammation	Early graft failure, cardiogenic shock, need for MCS or re-transplantation
Cardiac Allograft Vasculopathy (CAV)	Chronic vascular injury	Intimal hyperplasia due to immune and non-immune mechanisms	Progressive ischemia, heart failure, arrhythmias
Bacterial Infections	Gram-negative pathogens (*P. aeruginosa*, *Enterobacteriaceae*)	Sepsis-induced myocardial depression, systemic inflammation	Hemodynamic instability, graft dysfunction, septic shock
Viral Infections	Cytomegalovirus (CMV)	Direct myocardial injury; immune activation; promotion of CAV	Increased risk of rejection, graft loss, long-term CAV development
Fungal Infections	*Candida*, *Aspergillus* species	Invasive tissue infection, myocarditis, endocarditis	High morbidity/mortality, graft dysfunction, systemic illness
Parasitic Infections	*Toxoplasma gondii*	Reactivation or donor-derived transmission causing myocarditis	Myocardial necrosis, graft failure, systemic dissemination

Multiple mechanisms can contribute to graft dysfunction following orthotopic heart transplant, which may manifest early or develop as late complications. In the immediate post operative phase, PGD remains a leading cause of early mortality, occurring within the first 24 h post-transplant. PGD is characterized by severe biventricular dysfunction in the absence of identifiable secondary causes such as hyperacute rejection or surgical complications (13). Despite ongoing research, the precise pathophysiologic mechanisms underlying PGD remain incompletely understood.

CAV is the predominant cause of late graft loss and mortality following OHT. It is an accelerated form of coronary artery disease unique to transplant recipients, characterized by diffuse intimal hyperplasia affecting both epicardial and microvascular coronary vessels. CAV arises from a complex interplay of alloimmune injury, chronic inflammation, endothelial dysfunction, and traditional atherosclerotic risk factors. According to the International Society of Heart and Lung Transplantation registry, the prevalence of CAV is approximately 24% at five years and exceeds 50% by ten years post-transplantation (14).

Infectious etiologies represent a critical and often underestimated source of secondary graft dysfunction. Opportunistic infections are potentiated by long-term immunosuppression and can result in both direct myocardial injury and systemic inflammatory sequelae. Bacterial infections, particularly those involving Gram-negative organisms such as *Pseudomonas aeruginosa* and *Enterobacteriaceae*, frequently occur in the early postoperative period and are associated with sepsis-induced myocardial dysfunction and elevated mortality (15).

Among viral pathogens, cytomegalovirus (CMV) plays a central role in both infectious morbidity and immunologic injury to the graft. CMV infection is linked to an increased risk of acute rejection, allograft dysfunction, and progression of CAV. A recent multicenter study demonstrated that CMV infection in seropositive recipients was associated with increased graft loss and adverse clinical outcomes, although valganciclovir prophylaxis significantly mitigated these risks (16). Both early-onset and late-onset CMV infections have been implicated in long-term graft injury, underscoring the importance of sustained viral surveillance and individualized prophylactic strategies (17, 18).

Fungal infections, while less frequent, are associated with high morbidity and mortality due to their invasive nature. *Candida* and *Aspergillus* species can cause localized or disseminated infections, including endocarditis, mediastinitis, and myocarditis, all of which compromise graft integrity. Prompt recognition and antifungal therapy are critical to preserving graft function in these cases (19).

Additionally, protozoal infections such as *Toxoplasma gondii*, which are typically transmitted via donor organs, pose a unique risk, particularly in seronegative recipients. Reactivation in previously infected individuals or primary transmission in naïve recipients may lead to severe myocarditis and graft failure. As such, targeted screening and prophylaxis are recommended in high-risk populations (20).

The differential diagnosis for graft dysfunction following heart transplantation extends beyond rejection to include PGD, CAV and infectious etiologies, each of which contributes uniquely to allograft injury and recipient prognosis. Often an EMBx is performed to help rule out rejection as a reason for graft dysfunction. Meticulous screening, early detection, and individualized management strategies remain essential to improving long-term outcomes in heart transplant recipients.

## Traditional non-biopsy surrogate methods for rejection surveillance

Heart transplant rejection can be evaluated by several non-biopsy methods with each having its own distinct strength and limitations. Below we will discuss echocardiography, cardiac MRI, right heart catheterization (RHC), cardiac biomarkers, donor-specific antibodies (DSA), and non-HLA antibodies (Table 5).

**Table 5:** Comparison between traditional non-biopsy surrogate methods for rejection diagnosis.

Modality	Sensitivity	Specificity	Accessibility/Practical considerations	Clinical role
Echocardiography	Moderate	Moderate	Widely available	Routine surveillance tool which often prompts further testing if abnormal.
Cardiac MRI	High	Moderate-High	Limited availability	Provides tissue characterization and can non-invasively screen for rejection with high negative predictive value
Right Heart Catheterization (RHC)	N/A	N/A	Widely available at most transplant centers	Usually performed with biopsy. Abnormal RHC may trigger further evaluation for graft dysfunction.
Cardiac Biomarkers (Troponin, BNP)	Low (though sensitivity rises for severe rejection)	Low (non-specific elevations in cardiac stress)	High availability as a routine blood test	Triggers further diagnostics if abnormal but lacks specificity.
Donor-Specific Antibody	Moderate	Moderate	Requires specialized HLA lab	Persistent high levels suggests strong AMR risk.
Non-HLA Antibodies	Variable	Variable	Requires specialized labs	Could help explain AMR without DSA. Not part of the standard of care yet.
MMDx	High	High	High cost; requires an extra sample of endomyocardial tissue; limited prospective data	Improve sensitivity of EMBx when pre test probability is high from non invasive testing
miRNA	N/A	N/A	Not available for commercial use	Unclear

## Echocardiography

Echocardiography is a widely accessible, first-line tool that can be performed at the bedside to monitor allograft structure and function. A new decline in left ventricular systolic function or presence of diastolic dysfunction may raise the concern for rejection. However, neither clinical symptoms nor standard echocardiography are sufficiently sensitive to detect heart transplant rejection often necessitating frequent screening with endomyocardial biopsies (21). Advanced techniques such as speckle-tracking strain imaging have shown promise in improving sensitivity, but results have been variable and reproducibility in practice is a concern.

## Cardiac MRI

Cardiac MRI (cMRI) can be used to detect subtle graft injury through pulse sequences that create images that highlight different tissue characteristics such as fibrosis by T1 mapping and late gadolinium enhancement (LGE) and edema by T2-weighting. Literature review demonstrates that cMRI can identify acute rejection with high accuracy, with one meta-analysis reporting sensitivity ranging from 85% to 90% and specificity ranging from 70% to 85% using various markers (T1, T2, LGE) (22). Even though cMRI is non-invasive the study is less readily available and more costly than echocardiography.

## Right heart catheterization

Right heart catheterization (RHC) involves invasive measurement of intracardiac and pulmonary pressures and cardiac output. While this method cannot directly identify immune mediated rejection, it can detect indicators of significant graft failure with elevated filling pressures, low cardiac index, or high pulmonary vascular resistance.

## Cardiac biomarkers (troponin and natriuretic peptides)

Cardiac biomarkers tests are widely available, inexpensive, and non-invasive which makes them important surrogate markers for rejection diagnosis. In transplant patients, an unexplained increase in troponin and B-type natriuretic peptide can signal an issue with the graft and prompt further evaluation. It is important to note that diagnostic accuracy of these markers for rejection is limited due to low sensitivity and specificity for anything below severe rejection (23).

## Donor-specific antibodies

Donor-specific antibodies (DSA) are recipient antibodies that are directed against the donor's HLA antigens. The development of *de novo* DSA after transplant is a significant risk factor for antibody-mediated rejection (AMR) and is part of the current diagnostic criteria for AMR. As part of post-transplant surveillance, many transplant centers routinely monitor DSA levels in heart transplant recipients. Rising titers often prompt closer investigation or biopsy though it is important to note that not all DSA-positive patients have rejection. Hence the presence of DSA significantly increases suspicion for AMR (24) and could warrant further investigation.

## Non-HLA antibodies

Non-HLA antibodies are recipient antibodies against donor proteins other than HLA such as major histocompatibility complex class I chain-related antigens and other polymorphic antigens. Studies have shown that non-HLA antibodies can be associated with cardiac allograft rejection and graft dysfunction (25). The one drawback is that the overall sensitivity and specificity of any given non-HLA antibody for predicting rejection are not well established. It is a promising tool but requires further validation.

## Contemporary non-biopsy surrogate markers for rejection surveillance

### Gene expression profiling

Gene expression profiling (GEP) with the AlloMap® blood test (CareDx Inc, Brisbane, CA) was developed by comparing peripheral blood mononuclear cell RNA expression between rejection (ISHLT grade ≥3A) and non-rejection samples (26). The test analyzes peripheral blood for rates of expression of 11 genes involved in T cell and natural killer cell activation, recruitment, and trafficking, and yields a score ranging from 0 to 40 based on relative levels of gene expression. Higher scores are associated with higher grades of acute cellular rejection (ACR) with scores above 30 demonstrating a negative predictive value (NPV) of 99.6% for ISHLT grade ≥3A ACR (26), and scores ≥ 34 with a NPV of 98.1% to 100% as early as 2 months following transplant (27). Notably, GEP was not developed or validated for the detection of AMR.

The utility of GEP for noninvasive surveillance of ACR was explored in the IMAGE multicenter randomized controlled trial (28). IMAGE investigators randomly assigned 602 patients more than 6 months out from cardiac transplantation to be monitored for rejection using GEP vs. routine EMBx, and performed a noninferiority analysis comparing rates of a composite outcome of rejection with hemodynamic compromise, graft dysfunction due to other causes, death, or retransplantation (28). At median follow up of 19 months, patients monitored with GEP or routine EMBx had similar rates of the composite primary outcome and similar 2-year rates of death from any cause, with significantly fewer biopsies per person-year of follow up (28).

These results were followed by the single-center EIMAGE randomized controlled trial, evaluating the utility of GEP compared to EMBx from 2 to 6 months post-transplant (29). The primary end point was a composite of death, retransplant, rejection with hemodynamic compromise, or graft dysfunction at 18 months post-transplant. Investigators found that the composite primary end-point was similar between both arms and there was no significant difference in mean maximal intimal thickness by intravascular ultrasound among patients who underwent surveillance with GEP or EMBx beginning at 55 days post-transplant (29). The OAR study validated GEP across a multicenter cohort of 1504 low-risk heart transplant patients, noting that rates of acute rejection and death were low across the cohort of patients who underwent GEP surveillance, and interestingly that GEP scores did not correlate to rates of coronary allograft vasculopathy, malignancy, or non-cytomegalovirus infections (30).

These data led to the 2010 and 2023 ISHLT guidelines giving GEP a Class IIa (Level of Evidence: B) recommendation, and the European Society for Organ Transplantation (ESOT) consensus statement giving GEP a strong recommendation for risk stratification of ACR in low-risk heart transplant recipients (4, 31). Notably, GEP may be impacted by a number of external factors including corticosteroid administration (prednisone dose ≥20 mg daily), infections particularly with CMV, and leukopenia, and results must be interpreted in the context of these factors (4, 31, 32).

### Donor-derived cell-free DNA

Cell-free DNA are extracellular fragments of DNA that are released into the circulation from donor and recipient cells, and shotgun sequencing of purified DNA allows for identification and quantification of donor-derived cell-free DNA (dd-cfDNA) through single nucleotide polymorphisms (SNP) which vary between donor and recipient cells (4). A number of commercially available assays can be utilized to quantify the percentage of dd-cfDNA in the heart transplant recipient's blood, with higher levels indicating tissue injury and rejection (32). These include the AlloSure® (CareDx Inc, Brisbane, CA) assay utilizing a 405 SNP panel and the Prospera™ (Natera, Austin, TX) assay of 13,292 SNPs (33). While dd-cfDNA surveillance has not been compared to EMBx in a randomized controlled trial, a number of observational studies have demonstrated dd-cfDNA has a high negative-predictive value for ACR and AMR (31, 32).

The multicenter prospective D-OAR observational registry compared 2,199 dd-cfDNA samples with the AlloSure assay to EMBx across 740 patients, noting that patients with acute rejection had significantly higher dd-cfDNA levels than those without acute rejection, and dd-cfDNA levels below 0.2% had a 97% negative predictive value for acute rejection. Interestingly, dd-cfDNA levels additionally correlated with the presence of graft dysfunction, and were 3 times higher for patients with AMR as compared to ACR (34).

The subsequent GRAfT multicenter prospective study compared a research-grade dd-cfDNA assay using shotgun sequencing of 1,834 samples to 1,392 EMBx samples. Investigators found that median dd-cfDNA levels declined predictably by 28 days following transplant surgery and increased with acute rejection as compared to control samples. Moreover, dd-cfDNA levels increased 0.5–3.2 months prior to biopsy-proven acute rejection, and were higher for AMR compared to ≥ ACR. Investigators concluded that a 0.25% dd-cfDNA threshold had a 99% NPV for acute rejection and would safely eliminate 81% of EMBx (35).

Similar findings were reported from a 2-center study of the Prospera dd-cfDNA assay across 811 plasma samples from ≥ 28 days post-transplant. Patients with acute rejection had higher dd-cfDNA levels than controls, and patients with AMR had higher dd-cfDNA levels than those with ACR. This study also demonstrated that dd-cfDNA levels correlate with allograft dysfunction even in the absence of biopsy-proven rejection (36). The dd-cfDNA assays may be impacted by systemic infectious or inflammatory processes (which can increase recipient cell-free DNA and falsely decrease the percentage of dd-cfDNA), following endomyocardial biopsy, during pregnancy, or following multiorgan transplantation (10, 37). Recognizing this limitation, investigators have coupled the donor quantity score (DQS), an estimate of the total concentration of dd-cfDNA in plasma (genomic copies per milliliter cp/mL) with dd-cfDNA to improve the accuracy in detection of acute rejection (38).

Results from the two commercially available assays for dd-cfDNA, AlloSure and Prospera, have been compared with endomyocardial biopsy samples in retrospective analyses (33, 39). These analyses have demonstrated a high concordance rate of findings between the two assays, with a 39% sensitivity, 82%–84% specificity, and 98% negative predictive value for biopsy-proven acute rejection (33). There was no significant difference in accuracy of detection of acute rejection with the standard vs. the expanded SNP panel.

While GEP has a number of randomized trials supporting its utility, it is important to note that this study has only been validated for the detection of ACR. GEP data can be coupled with dd-cfDNA to further risk stratify, as dd-cfDNA assays have been validated for evaluation of AMR. GEP currently only has one commercially available assay (AlloMap), whereas dd-cfDNA may be evaluated using a number of commercially available tests (Prospera, AlloSure) or using a combination GEP and dd-cfDNA assay (HeartCare, combining AlloMap GEP assay and AlloSure dd-cfDNA assay) (10). These two assays provide complementary information regarding a patient's risk for ACR and AMR, as well as degree of myocardial injury (40), and are increasingly being used to reduce surveillance EMBx in the post-transplant population.

The SHORE registry evaluated the impact of combined GEP and dd-cfDNA testing in contemporary post-heart transplant patients enrolled between 2018 and 2021. Investigators demonstrated that dual molecular testing demonstrated improved performance for ACR detection compared to either test alone, with dual positivity associated with a high likelihood ratio of ACR detection (Table 6). Furthermore, dual molecular testing was associated with lower biopsy rates over time, overall excellent survival in a population representative of the current US heart transplant population, and an overall low incidence of graft dysfunction (37).

**Table 6:** Incidence of acute cellular rejection (ACR) per data from the SHORE registry (37) based on results of combination gene expression profiling (GEP; alloMap®, careDx Inc., Brisbane, CA) and dd-cfDNA assays (alloSure®, careDx Inc., Brisbane, CA).

		AlloSure ≥ 0.2% 28 days post-transplant	
		Negative	Positive
AlloMap ≥30 55 days to 6-months post transplant ≥34 after 6 months post-transplant	Negative	1.5% incidence of ACR Low likelihood of rejection.	4.3% incidence of ACR Consider etiologies of graft injury, including AMR, CAV, and trauma.
	Positive	1.9% incidence of ACR Consider systemic infectious or inflammatory etiologies.	9.2% incidence of ACR Strong likelihood of rejection.

AlloMap testing was defined as positive with a total level ≥30 between 55 days and 6 months post-transplant, or a level ≥ 34 after 6 months post-transplant. AlloSure testing was defined as positive at ≥0.2% at any time post-transplant. The incidence of associated ACR ranged from 1.5% to 9.2%, and correlated with noninvasive testing measurements. Possible etiologies of false positive GEP testing include systemic infectious or inflammatory processes, and possible etiologies of false positive dd-cfDNA testing include allograft injury, AMR, CAV, and trauma including endomyocardial biopsy. Notably, rates of antibody-mediated rejection (AMR) were not analyzed based off of SHORE registry data.

It remains unclear whether either GEP or dd-cfDNA testing confers additive prognostic value to contemporary surveillance methodologies, and whether either or both of these techniques may be used to guide immunosuppression strategies. The upcoming DEFINE-HT prospective observational study will evaluate the association of dd-cfDNA with biopsy-diagnosed rejection and clinical outcomes across 150 patients 1 year post heart transplant (NCT05309382). Results are awaited from the upcoming DETECT trial (NCT005081739) in which patients are randomized to traditional EMBx vs. noninvasive surveillance strategies to evaluate post-heart transplant outcomes, as well as the MOSAIC trial (NCT05459181) in which dd-cfDNA levels are used to guide immunosuppression strategy (10). These trials will inform the real-world clinical utility of GEP and dd-cfDNA analysis to safely minimize EMBx utilization for the detection of acute rejection, and potentially guide immunosuppression as well as inform prognosis.

## MicroRNA and mRNA in tissue

While histologic assessment has remained the gold standard in diagnosing allograft rejection for decades, the aforementioned limitations of the endomyocardial biopsy sample (namely human subjectivity in sample adjudication) and their consequent implications on care delivery and healthcare expenditure have behooved investigators to seek to develop alternative more objective invasive, tissue-based rejection surveillance modalities (41). Borrowing from the world of oncology, where molecular phenotyping of tissue has long been utilized to better characterize disease and guide treatment, there has been an ever-present focus on studying both messenger RNA and microRNA expression of biopsy samples as an adjunct to traditional rejection surveillance methods.

One such system, known as the Molecular Microscope® Diagnostic System (MMDx), offers a standardized, and objective adjunct methodology for classifying and grading rejection. Using microarray technology and multi-archetype analysis, mRNA expression of a biopsy sample are compared to a reference set of biopsies, identifying specific Rejection-associated transcripts (RATs) and characterizing the sample with regards to probability of distinct archetypes T Cell Mediated Rejection (TCMR), Antibody mediated rejection (ABMR) and other disease/non rejection injury states (42). Biopsy samples are immediately placed in a proprietary solution (RNAlater), mRNA is extracted and undergoes hybridization and microarray analysis is performed, ultimately generating a CEL file representative of 19,462 unique genes. This CEL file undergoes processing via proprietary unsupervised machine learning algorithm-based MMDx software with results available 48 h later. Scores are generated for each archetype from 0 to 1, with the highest archetype score used to assign each biopsy to a particular archetype cluster." (41).

Initially validated for kidney transplant recipients, investigators of the INTERHEART study and its subsequent analyses sought to evaluate the overlap of RATs between the heart and kidney transplant population and the overall efficacy of MMDx in characterizing rejection in endomyocardial biopsy specimens (41). In the initial iteration of the study, scores were relegated to the samples, delineating them into three RAT-based archetypes of TCMR, ABMR and no rejection (43). There was larger discordance with histology in MMDx-Heart samples, compared to their MMDx-Kidney counterparts across all three archetypes, and was noted to be particularly worse with higher grades of TCMR. Further iterations of the study with more biopsy samples resulted in the construction of the fourth and fifth archetypes, namely “injury” and “minor rejection” respectively, as well as binary molecular rejection classifier scores to better characterize the spectrum of rejection (41, 43). The contemporary MMDx report includes 2 scores based on the 3 and 4-archetype models respectively, as well as molecular classifier scores and expression values for implicated gene sets, culminating into a curated interpretation of the probability of rejection/injury of a given biopsy sample (41). Independence from relying on histology allows the MMDx platform to serve as an adjunct, more objective measure of rejection, less prone to inter-operator variability and sampling error.

While long term follow up from the INTERHEART study may be limited, contemporary observational data continues to support the utility of MMDx in helping reclassify rejection and assist in predicting patient outcomes (43).

In a single center retrospective analysis, Alam et al. demonstrated that while there continues to be relatively high degree of concordance between EMBx and MMDx samples in characterizing rejection (86%), cases of discordance where MMDx resulted positive and rejection was suggested by means of other non invasive adjunctive clinical features (i.e., DSA, ddcfDNA), resulted in treatment of 5 patients with BNR who remained negative on subsequent EMBx and 3 patients who went on to demonstrate features of rejection on biopsy, suggesting the utility of MMDx to reclassify rejection at an earlier phase (44). Furthermore, although Valledor et al. similarly demonstrated a high degree of concordance between EMBx and MMDx rejection classification (76.8% agreement), 79/95 samples were noted to be negative for histologic rejection but positive for MMDx rejection. In 73.4% of cases of discordance between EMBx and MMDx, the immunosuppression strategy was altered. One year survival for all patients for whom MMDx resulted in a change in immunosuppression strategy, was found to be 87% compared to 78.6% in patients with a positive MMDx but without a change in their immunosuppression regimen (44), demonstrating the real world implications of the MMDx system.

Limitations of the system include cost, logistics and prospective data to guide treatment. At $3,159 per test, MMDx remains one of the most costly molecular genomic based rejection assessment modalities (45). The need for a separate endomyocardial biopsy specimen to be immediately placed into RNAlater solution as well multiple steps of mRNA post processing also add additional steps to work flow. Additionally there is limited prospective data to help guide in nuanced scenarios such as that of MMDx positive, biopsy negative rejection (43).

In addition to mRNA based analyses, micro RNA (miRNA) - with its important role in regulating intricate genetic networks and protein expression in multiple immune conditions, has been utilized in investigating non invasive and invasive tissue-derived assays as viable adjuncts in the evaluation for allograft rejection. Specific circulating microRNA motifs have shown some promise in predicting rejection across the spectrum of solid organ transplants, whether it be miRNA-15B, miRNA-16, miRNA-103 A, miRNA-106 A, and miRNA-107 in renal transplant, miR-21 in lung transplant or miR-155-5p, miR-181a-5p and miR-122-5p in liver transplant in observational studies (46). However, with regards to cardiac allograft rejection, Coutance et al. demonstrated in a multicenter prospective longitudinal study that previously identified clinically relevant miRNA signatures (10a, 92a, 155) in fact had no association with allograft rejection with the study stopping early for futility, further demonstrating that the role of circulating miRNA in predicting cardiac allograft rejection remains unclear (47). There does however appear to increasing support in the literature for the feasibility of a tissue based miRNA based assay akin to MMDx for mRNA as demonstrated in the work of Novakova et al., where 11 distinct tissue based miRNA motifs were significantly dysregulated in the presence of allograft rejection (48). To date there is no tissue derived miRNA-based diagnostic testing system that is commercially available. More data is needed to assess the feasibility and cost-effectiveness of these strategies as adjuncts to the gold standard, histologic and immunopathologic assessment of endomyocardial biopsy samples.

## The future of rejection surveillance monitoring

While the definitions of ACR and AMR put forth by the ISHLT are comprehensive in their own right, there are entities within the spectrum of rejection that are not well characterized by these definitions. Biopsy negative rejection (BNR) was previously described as hemodynamic compromise in the absence of ACR in the era before standardized definitions of AMR were adopted. BNR, more recently described as a decrement in LVEF <45% without evidence of ACR or AMR (49), carries significant implications on transplant outcomes including 5 year survival, development of CAV and NF-MACE, irrespective of severity or time (49, 50). With the advent of high fidelity adjunctive rejection surveillance strategies and their ability to characterize rejection with high sensitivity and specificity (i.e., positive MMDx and/or positive cfDNA + positive GEP in setting of negative biopsy, high DSAs with negative biopsy), it appears the definition of BNR may once again need revising.

A recent analysis of the SHORE registry focusing on AMR with respect to degree of dd-cfDNA elevation, graft function on echo and presence or absence of DSA found: AMR occurred in 1.1% of biopsies with normal graft function and no DSAs vs. 20.4% of biopsies with known DSA and graft dysfunction. In patients with neither DSA nor graft dysfunction, the incidence of AMR was 0.7% for dd-cfDNA levels <0.20%, 1.2% for levels between 0.20% and 0.49%, and 6.7% for dd-cfDNA levels ≥0.50%. In patients with known DSA but no graft dysfunction, the incidence of AMR was 1.4% for dd-cfDNA levels <0.20%, 4.8% for levels between 0.20% and 0.49%, and 15.5% for dd-cfDNA levels ≥0.50% (51). While the future appears bright for the world of non invasive rejection surveillance especially with the above findings, the likelihood that the EMBx will be outright replaced appears to be low. With the advent of commercially available GEP and cfDNA based assays the average number of biopsies in year one after transplant has dropped considerably in centers across the nation (10). Notwithstanding, the number of biopsies in year one have more often than not decreased from double digits to single digits, however they have not been phased out entirely, nor will it be anytime soon – if ever. For centers shifting to heavily relying on non-invasive surveillance, positive results (i.e., GEP and/or cfDNA) invariably lead to a confirmatory EMBx, to consider treatment options. Ultimately, the transplant community may choose to relegate the EMBx to where it should perhaps belong - as a diagnostic tool when noninvasive surveillance modalities indicate its necessity.

With the advent of neural networks, deep learning and machine learning-based algorithms, artificial intelligence is poised to revolutionize the diagnosis and treatment of allograft rejection. There already appears to be growing body of evidence in the transplant literature highlighting the utility of AI in reclassifying and recharacterizing rejection in allograft biopsy samples, including but not limited to the works of Zhang et al., where a masked region based neural network was used to compare native kidney biopsies to those post transplant demonstrating one year graft loss prediction superior to that of the Banff classification system (52). Additionally, the Banff Automation System, an automated algorithm based on the Banff criteria for renal allograft rejection that includes histologic lesions and scores, C4d staining, presence of DSA and available molecular markers has demonstrated significant additive discriminative ability with cases previously classified as no rejection reclassified as rejection exhibiting worse graft survival (HR = 6.4, 95% CI: 3.9–10.6, *p* value < 0.0001) (53). In the heart transplant literature, Glass et al. demonstrated the utility of supervised machine learning in identifying histologic ACR on EMBx with 99% validation accuracy (54) as well as histologic pAMR-H with >99% validation accuracy (55). Similarly, Arabyarmohammadi et al, utilizing a proof-of-principle machine learning pipeline named “the cardiac allograft rejection evaluator” (CARE) sought to highlight the predictive power of morphologic data in endomyocardial specimens (namely lymphocytes and stroma) not routinely included in the standard ISHLT guidelines (56). Each of 2,900 biopsy specimens, were stratified based on rejection grade (high vs. low) and clinical trajectory (evident vs. silent) and 370 morphologic features were identified via use of a digital pathology imaging analysis pipeline, ultimately yielding 5 unique features that correlated with rejection trajectories (namely endocardial stromal solidity, Interstitial stroma eccentricity, Lymphocyte foci count, Lymphocyte area ratio, Lymphocyte foci count in myocardium). In the world of non invasive ancillary diagnostics, Adedinsewo et al. demonstrated the utility of supervised deep learning algorithm in detecting ECG changes that correlate with moderate-severe ACR with a retrospective AUC of 0.84 (95% confidence interval: 0.78–0.90) and Sensitivity of 95% (CI: 75%–100%) and prospective AUC of 0.78 (CI 0.61–0.96) and sensitivity of 100% (CI: 16%–100%) (45).

In addition to use of AI in the the mitigation of interobserver variability and adjunctive utility in the review of primary clinical data (i.e histopathology, ancillary testing such as ECG, TTE, cMRI etc.), the formation of multimodal neural networks that can compute using multiparametric data sets to better risk stratify for rejection and further curtail precision treatment offers a powerful new frontier. One ongoing study that seeks to elucidate just that is the “Precision Medicine in the Management of Heart Transplant Recipients” a prospective single center randomized controlled trial set to be completed in 2027, that seeks to investigate the longitudinal impact of integration of clinical, molecular, imaging and histologic data on risk of rejection, graft dysfunction and infection (57).

The way of the future appears multimodal- striking a balance between various invasive and non-invasive rejection surveillance modalities, informed and enhanced by artificial intelligence, to optimally characterize the state of rejection in patients and formulate unique treatment strategies.

## References

[1] Billingham ME Cary NR Hammond ME Kemnitz J Marboe C McCallister HA A working formulation for the standardization of nomenclature in the diagnosis of heart and lung rejection: heart rejection study group. The international society for heart transplantation. J Heart Transplant. (1990) 9(6):587–93. 2277293

[2] Stewart S Winters GL Fishbein MC Tazelaar HD Kobashigawa J Abrams J Revision of the 1990 working formulation for the standardization of nomenclature in the diagnosis of heart rejection. J Heart Lung Transplant. (2005) 24(11):1710–20. 10.1016/j.healun.2005.03.019 16297770

[3] Colvin MM Cook JL Chang P Francis G Hsu DT Kiernan MS Antibody-mediated rejection in cardiac transplantation: emerging knowledge in diagnosis and management: a scientific statement from the American Heart Association. Circulation. (2015) 131(18):1608–39. 10.1161/CIR.0000000000000093 25838326

[4] Velleca A Shullo MA Dhital K Azeka E Colvin M DePasquale E The international society for heart and lung transplantation (ISHLT) guidelines for the care of heart transplant recipients. J Heart Lung Transplant. (2023) 42(5):e1–e141. 10.1016/j.healun.2022.10.015 37080658

[5] Melvin KR Mason JW. Endomyocardial biopsy: its history, techniques and current indications. Can Med Assoc J. (1982) 126(12):1381–6. ; PMCID: PMC1863164. 7044509 PMC1863164

[6] Farcas AO Stoica MC Maier IM Maier AC Sin AI. Heart transplant rejection: from the endomyocardial biopsy to gene expression profiling. Biomedicines. (2024) 12(8):1926. 10.3390/biomedicines12081926 39200392 PMC11351478

[7] From AM Maleszewski JJ Rihal CS. Current status of endomyocardial biopsy. Mayo Clin Proc. (2011) 86(11):1095–102. 10.4065/mcp.2011.0296 22033254 PMC3203000

[8] Oh KT Mustehsan MH Goldstein DJ Saeed O Jorde UP Patel SR. Protocol endomyocardial biopsy beyond 6 months-it is time to move on. Am J Transplant. (2021) 21(2):825–9. 10.1111/ajt.16128 32515104

[9] Coutance G Kransdorf E Aubert O Bonnet G Yoo D Rouvier P Clinical prediction model for antibody-mediated rejection: a strategy to minimize surveillance endomyocardial biopsies after heart transplantation. Circ Heart Fail. (2022) 15(10):e009923. 10.1161/CIRCHEARTFAILURE.122.009923 36200456

[10] Holzhauser L DeFilippis EM Nikolova A Byku M Contreras JP De Marco T The End of endomyocardial biopsy?: a practical guide for noninvasive heart transplant rejection surveillance. JACC Heart Fail. (2023) 11(3):263–76. 10.1016/j.jchf.2022.11.002 36682960

[11] Ludhwani D Abraham J Sharma S Kanmanthareddy A. Heart Transplantation Rejection. Treasure Island, FL: StatPearls (2025. ineligible companies. Disclosure: Joseph Abraham declares no relevant financial relationships with ineligible companies. Disclosure: Sanjeev Sharma declares no relevant financial relationships with ineligible companies. Disclosure: Arun Kanmanthareddy declares no relevant financial relationships with ineligible companies. 30725742

[12] Alam A Wilcox JE Hall SA. The traditional endomyocardial biopsy: opportunities to rethink its role as the gold standard. J Card Fail. (2023) 29(8):1225–7. 10.1016/j.cardfail.2021.05.029 34242781

[13] Alam AH Lee CY Kanwar MK Moayedi Y Bernhardt AM Takeda K Current approaches to optimize the donor heart for transplantation. J Heart Lung Transplant. (2025) 44(4):672–80. 10.1016/j.healun.2024.12.001 39730081

[14] Chambers DC Cherikh WS Harhay MO Hayes D Jr Hsich E Khush KK The international thoracic organ transplant registry of the international society for heart and lung transplantation: thirty-sixth adult heart transplantation report - 2019; focus theme: donor and recipient size match. J Heart Lung Transplant. (2019) 38(10):1056–66. 10.1016/j.healun.2019.08.004 31548031 PMC6816343

[15] Neofytos D Stampf S Hoessly LD D'Asaro M Tang GN Boggian K Bacteremia during the first year after solid organ transplantation: an epidemiological update. Open Forum Infect Dis. (2023) 10(6):ofad247. 10.1093/ofid/ofad247 37323422 PMC10267299

[16] Gardiner BJ Bailey JP Percival MA Morgan BA Warner VM Lee SJ Incidence and severity of cytomegalovirus infection in seropositive heart transplant recipients. Clin Transplant. (2023) 37(6):e14982. 10.1111/ctr.14982 36988473 PMC10909407

[17] Afzal A Alam A van Zyl JS Zafar H Felius J Hall SA Observed elevated donor-derived cell free DNA in orthotopic heart transplant recipients without clinical evidence of rejection. Clin Transplant. (2022) 36(3):e14549. 10.1111/ctr.14549 34863042 PMC9286598

[18] Alam AH Van Zyl J Shakoor HI Farsakh D Abdelrehim AB Maliakkal N The impact of active cytomegalovirus infection on donor-derived cell-free DNA testing in heart transplant recipients. Clin Transplant. (2024) 38(3):e15287. 10.1111/ctr.15287 38477177

[19] Kabir V Maertens J Kuypers D. Fungal infections in solid organ transplantation: an update on diagnosis and treatment. Transplant Rev (Orlando). (2019) 33(2):77–86. 10.1016/j.trre.2018.12.001 30579665

[20] Mamizadeh M Maleki F Mohammadi MR Shamsi L Asghari A Pouryousef A. Seroprevalence and risk factors for toxoplasma gondii infection in solid organ transplant patients: a global systematic review and meta-analysis. Parasite Epidemiol Control. (2025) 29:e00421. 10.1016/j.parepi.2025.e00421 40129460 PMC11932682

[21] Butler CR Savu A Bakal JA Toma M Thompson R Chow K Correlation of cardiovascular magnetic resonance imaging findings and endomyocardial biopsy results in patients undergoing screening for heart transplant rejection. J Heart Lung Transplant. (2015) 34(5):643–50. 10.1016/j.healun.2014.12.020 25934478

[22] Han D Miller RJH Otaki Y Gransar H Kransdorf E Hamilton M Diagnostic accuracy of cardiovascular magnetic resonance for cardiac transplant rejection: a meta-analysis. JACC Cardiovasc Imaging. (2021) 14(12):2337–49. 10.1016/j.jcmg.2021.05.008 34274269

[23] Hayward C. Cardiac allograft injuries: a review of approaches to a common dilemma, with emphasis on emerging techniques. Int J Heart Fail. (2022) 4(3):123–35. 10.36628/ijhf.2021.0042 36262796 PMC9383355

[24] Goldberg JF Mehta A Bahniwal RK Agbor-Enoh S Shah P. A gentler approach to monitor for heart transplant rejection. Front Cardiovasc Med. (2024) 11:1349376. 10.3389/fcvm.2024.1349376 38380175 PMC10876874

[25] Butler CL Hickey MJ Jiang N Zheng Y Gjertson D Zhang Q Discovery of non-HLA antibodies associated with cardiac allograft rejection and development and validation of a non-HLA antigen multiplex panel: from bench to bedside. Am J Transplant. (2020) 20(10):2768–80. 10.1111/ajt.15863 32185871 PMC7494540

[26] Deng MC Eisen HJ Mehra MR Billingham M Marboe CC Berry G Noninvasive discrimination of rejection in cardiac allograft recipients using gene expression profiling. Am J Transplant. (2006) 6(1):150–60. 10.1111/j.1600-6143.2005.01175.x 16433769

[27] Crespo-Leiro MG Stypmann J Schulz U Zuckermann A Mohacsi P Bara C Clinical usefulness of gene-expression profile to rule out acute rejection after heart transplantation: cARGO II. Eur Heart J. (2016) 37(33):2591–601. 10.1093/eurheartj/ehv682 26746629 PMC5015661

[28] Pham MX Teuteberg JJ Kfoury AG Starling RC Deng MC Cappola TP Gene-expression profiling for rejection surveillance after cardiac transplantation. N Engl J Med. (2010) 362(20):1890–900. 10.1056/NEJMoa0912965 20413602

[29] Kobashigawa J Patel J Azarbal B Kittleson M Chang D Czer L Randomized pilot trial of gene expression profiling versus heart biopsy in the first year after heart transplant: early invasive monitoring attenuation through gene expression trial. Circ Heart Fail. (2015) 8(3):557–64. 10.1161/CIRCHEARTFAILURE.114.001658 25697852

[30] Moayedi Y Foroutan F Miller RJH Fan CS Posada JGD Alhussein M Risk evaluation using gene expression screening to monitor for acute cellular rejection in heart transplant recipients. J Heart Lung Transplant. (2019) 38(1):51–8. 10.1016/j.healun.2018.09.004 30352779

[31] Nikolova A Agbor-Enoh S Bos S Crespo-Leiro M Ensminger S Jimenez-Blanco M European Society for organ transplantation (ESOT) consensus statement on the use of non-invasive biomarkers for cardiothoracictransplant rejection surveillance. Transpl Int. (2024) 37:12445. 10.3389/ti.2024.12445 38962472 PMC11221358

[32] Kobashigawa J Hall S Shah P Fine B Halloran P Jackson AM The evolving use of biomarkers in heart transplantation: consensus of an expert panel. Am J Transplant. (2023) 23(6):727–35. 10.1016/j.ajt.2023.02.025 36870390 PMC10387364

[33] Rodgers N Gerding B Cusi V Vaida F Tada Y Morris GP Comparison of two donor-derived cell-free DNA tests and a blood gene-expression profile test in heart transplantation. Clin Transplant. (2023) 37(6):e14984. 10.1111/ctr.14984 37036133 PMC10330254

[34] Khush KK Patel J Pinney S Kao A Alharethi R DePasquale E Noninvasive detection of graft injury after heart transplant using donor-derived cell-free DNA: a prospective multicenter study. Am J Transplant. (2019) 19(10):2889–99. 10.1111/ajt.15339 30835940 PMC6790566

[35] Agbor-Enoh S Shah P Tunc I Hsu S Russell S Feller E Cell-Free DNA to detect heart allograft acute rejection. Circulation. (2021) 143(12):1184–97. 10.1161/CIRCULATIONAHA.120.049098 33435695 PMC8221834

[36] Kim PJ Olymbios M Siu A Wever Pinzon O Adler E Liang N A novel donor-derived cell-free DNA assay for the detection of acute rejection in heart transplantation. J Heart Lung Transplant. (2022) 41(7):919–27. 10.1016/j.healun.2022.04.002 35577713 PMC9670834

[37] Khush K Hall S Kao A Raval N Dhingra R Shah P Surveillance with dual noninvasive testing for acute cellular rejection after heart transplantation: outcomes from the surveillance HeartCare outcomes registry. J Heart Lung Transplant. (2024) 43(9):1409–21. 10.1016/j.healun.2024.05.003 38759766

[38] Kim PJ Olympios M Sideris K Tseliou E Tran TY Carter S A two-threshold algorithm using donor-derived cell-free DNA fraction and quantity to detect acute rejection after heart transplantation. Am J Transplant. (2025) 25(9):1895–905. 10.1016/j.ajt.2025.07.1920 40334845 PMC12435761

[39] Hsi B Van Zyl J Alam K Shakoor H Farsakh D Alam A Tale of two assays: comparison of modern donor-derived cell-free DNA technologies. JHLT Open. (2024) 4:100090. 10.1016/j.jhlto.2024.100090 40144271 PMC11935440

[40] Halloran PF Reeve J Mackova M Madill-Thomsen KS Demko Z Olymbios M Comparing plasma donor-derived cell-free DNA to gene expression in endomyocardial biopsies in the trifecta-heart study. Transplantation. (2024) 108(9):1931–42. 10.1097/TP.0000000000004986 38538559 PMC11335077

[41] Halloran PF Madill-Thomsen KS. The molecular microscope diagnostic system: assessment of rejection and injury in heart transplant biopsies. Transplantation. (2023) 107(1):27–44. 10.1097/TP.0000000000004323 36508644

[42] Madill-Thomsen KS Halloran PF. Precision diagnostics in transplanted organs using microarray-assessed gene expression: concepts and technical methods of the molecular microscope(R) diagnostic system (MMDx). Clin Sci (Lond). (2024) 138(11):663–85. 10.1042/CS20220530 38819301 PMC11147747

[43] Mehlman Y Valledor AF Moeller C Rubinstein G Lotan D Rahman S The utilization of molecular microscope in management of heart transplant recipients in the era of noninvasive monitoring. Clin Transplant. (2023) 37(12):e15131. 10.1111/ctr.15131 37897211

[44] Alam A Van Zyl J Paul Milligan G Michelle McKean S Patel R Anne Hall S. Evolving the surveillance and workup of heart transplant rejection: a real-world analysis of the molecular microscope diagnostic system. Am J Transplant. (2022) 22(10):2443–50. 10.1111/ajt.17087 35514138

[45] Adedinsewo D Hardway HD Morales-Lara AC Wieczorek MA Johnson PW Douglass EJ Non-invasive detection of cardiac allograft rejection among heart transplant recipients using an electrocardiogram based deep learning model. Eur Heart J Digital Health. (2023) 4(2):71. 10.1093/ehjdh/ztad001 PMC10039431 36974261

[46] Yu S Lu J. MicroRNAs in transplant rejection: emerging roles in immune regulation and applications. Transpl Immunol. (2025) 90:102222. 10.1016/j.trim.2025.102222 40107626

[47] Coutance G Racapé M Baudry G Lécuyer L Roubille F Blanchart K Validation of the clinical utility of microRNA as noninvasive biomarkers of cardiac allograft rejection: a prospective longitudinal multicenter study. J Heart Lung Transplant. (2023) 42(11):1505–9. 10.1016/j.healun.2023.07.010 37487804

[48] Nováková T Macháčková T Novák J Hude P Godava J Žampachová V Identification of a diagnostic set of endomyocardial biopsy microRNAs for acute cellular rejection diagnostics in patients after heart transplantation using next-generation sequencing. Cells. (2019) 8(11):1400. 10.3390/cells8111400 31698874 PMC6912472

[49] Tang Z Kobashigawa J Rafiei M Stern LK Hamilton M. The natural history of biopsy-negative rejection after heart transplantation. J Transplant. (2013) 2013:236720. 10.1155/2013/236720 24490053 PMC3893856

[50] Balakrishnan K Ratnagiri R Rao S Tungaturu M. Limiting the number of endomyocardial biopsies does not impact one year survival following heart transplantation. J Heart Lung Transplant. (2016) 35(4):S210. 10.1016/j.healun.2016.01.590

[51] Kim PJ Alam AH Teuteberg JJ Khush KK Pinney SP Cheng RK Donor-Derived cell-free DNA in antibody-mediated rejection: an analysis of the surveillance HeartCare outcomes registry. JACC Heart Fail. (2025) 14(1):102716. 10.1016/j.jchf.2025.102716 41123513 PMC12719789

[52] Mouhamad AM Lakkis Zoe S Ansari Zuhayr A Cherukuri Aravind R Abou-Daya Khodor I. The transformative potential of artificial intelligence in solid organ transplantation. Front Transpl. (2024) 3:1361491. 10.3389/frtra.2024.1361491 PMC11235281 38993779

[53] Reeve J Böhmig GA Eskandary F Einecke G Gupta G Madill-Thomsen K Generating automated kidney transplant biopsy reports combining molecular measurements with ensembles of machine learning classifiers. Am J Transplant. (2019) 19(10):2719–31. 10.1111/ajt.15351 Erratum in: Am J Transplant. 2020 Feb;20(2):620-621. doi: 10.1111/ajt.15770. 30868758

[54] Glass C Davis R Xiong B Dov D Glass M. The use of artificial intelligence (AI) machine learning to determine myocyte damage in cardiac transplant acute cellular rejection. J Heart Lung Transplant. (2020) 39(4):S59. 10.1016/j.healun.2020.01.1250

[55] Glass M Ji Z Davis R Pavlisko EN DiBernardo L Carney J A machine learning algorithm improves the diagnostic accuracy of the histologic component of antibody mediated rejection (AMR-H) in cardiac transplant endomyocardial biopsies. Cardiovasc Pathol. (2024) 72:107646. 10.1016/j.carpath.2024.107646 38677634

[56] Arabyarmohammadi S Yuan C Viswanathan VS Lal P Feldman MD Fu P Failing to make the grade: conventional cardiac allograft rejection grading criteria are inadequate for predicting rejection severity. Circ Heart Fail. (2024) 17(2):e010950. 10.1161/CIRCHEARTFAILURE.123.010950 38348670 PMC10940208

[57] Precision Medicine in the Management of Heart Transplant Recipients. Clinicaltrials.gov. (n.d.). Available online at: https://clinicaltrials.gov/study/NCT06774794 (Accessed July 29, 2025).

